# Casticin Inhibits Osteoclastogenesis via NF-κB/BCL-2 Signaling Pathway

**DOI:** 10.4014/jmb.2409.09009

**Published:** 2025-02-13

**Authors:** An Huang, Zhiping Gu, Jiahao Jin, Tao Nie

**Affiliations:** 1 Orthopedic Hospital, Postdoctoral Innovation Practice Bace, The First Affiliated Hospital, Jiangxi Medical College, Nanchang University, Nanchang 330006, P.R. China; 2 Orthopedic Hospital, The First Affiliated Hospital, Jiangxi Medical College, Nanchang University, Nanchang, P.R. China

**Keywords:** Casticin, osteoclasts, NF-κB, BCL-2, signal pathway

## Abstract

This study investigates the effects of casticin on osteoclastogenesis, aiming to elucidate its underlying mechanisms for potential clinical applications. We assessed the cytotoxicity of casticin using a CCK assay in RAW 264.7cell (murine cell line, from ATCC), which differentiate into osteoclasts upon RANKL treatment. Various concentrations (0.125, 0.25, 0.50 μM) were tested to establish a dose-dependent response. The effects of casticin on osteoclast differentiation and actin filament organization were evaluated through TRAP and F-actin staining. Additionally, qPCR and Western blot analyses were performed to assess gene expression. Concentrations exceeding 1.00 μM caused significant cytotoxicity. Notably, casticin at 0.50 μM significantly inhibited osteoclast differentiation and function, reducing marker gene expression, including c-FOS, NFATc1, CtsK, and MMP-9. Furthermore, casticin decreased phosphorylation levels of NF-κB and IκBα and downregulated BCL-2 expression. Our findings highlight casticin's potent regulatory effects on osteoclasts via the NF-κB/BCL-2 signaling pathways, suggesting potential therapeutic applications in bone-related disorders.

## Introduction

Osteoporosis affects over 200 million people worldwide[1], characterized by low bone mass and structural deterioration primarily attributed to osteoclastogenesis [2, 3]. Bisphosphonates are the mainstay for osteoporosis treatment [4]. However, oral bisphosphonates cannot be applied to patients with esophageal abnormalities, vitamin D deficiency, hypocalcemia, hypersensitivity, and kidney failure. Besides, intravenous bisphosphonates may cause acute phase reactions [5]. Due to ongoing concerns about treatment toxicity and limitations, research is necessary to develop novel therapies for osteoporosis.

Traditional Chinese medicine (TCM) is valued for its high therapeutic potential, cost-effectiveness, and minimal toxicity, making it a promising complementary and alternative approach to enhance both survival rates and the quality of life in osteoporosis patients [5]. Many TCMs are known to contain phytoestrogens, which exhibit estrogen-like effects and can help preserve bone mass while inhibiting osteoclast activity [6]. Research has demonstrated that phytoestrogens, such as polymethoxyflavonoid compounds, possess the ability to restrain osteoclastogenesis (differentiation and function), bone resorption, and even reverse femoral bone mass loss in ovariectomized mice [7]. One such TCM is Manjingzi, derived from the dried and mature fruit of the single leaf Manjingzi or Manjingzi of the Verbenaceae family [8]. Manjingzi is known for its therapeutic properties in dispersing wind-heat and clearing the head. Casticin, a key active ingredient in Manjingzi, belongs to the category of polymethoxyflavones. Casticin is characterized by its multifaceted properties, including anti-tumor, antioxidant, anti-inflammatory, and immunosuppressive effects. Research has shown that casticin effectively inhibits the activity of mouse peritoneal macrophages, induces apoptosis in monocyte macrophages (RAW264.7 cells), and hinders the differentiation of RAW264.7 cells into osteoclasts [3]. However, the precise underlying mechanism by which casticin regulated the differentiation of RAW264.7 cells remains to be fully elucidated.

The OPG/RANKL/RANK pathway is critical for regulating osteoclast formation, with RANKL promoting osteoclast activity and OPG inhibiting it through competitive binding to RANK, which triggered the action of TRAF6/NF-κB/NFATc1 signaling pathway, leading to enhanced osteoclast formation [9]. As a downstream effector molecule of NF-κB [10, 11]. B-cell lymphoma-2 (BCL-2) was up-regulated in osteoclasts [12]. Conversely, osteoclast numbers decreased, extending their lifespan and resulting in increased bone mass in BCL-2 gene knockout mice, [10]. Notably, in specific osteoporotic conditions like postmenopausal osteoporosis, abnormally high BCL-2 expression coincided with the abnormal activation of the NF-κB signaling pathway. Furthermore, the reduction in estrogen levels can trigger this aberrant activation, influencing osteoblast proliferation and differentiation [13]. In addition, phytoestrogens can be classified according to their institutional characteristics into isoflavones, flavonoids, lignans, etc. casticin belongs to flavonoids, so it has weak estrogen effect [14]. Considering the structural similarity between Casticin and estrogen, We hypothesized that Casticin may regulate the differentiation of RAW264.7 cells through NF-κB signaling pathway.NF-κB signaling pathwayTo test our hypothesis, we initially assessed the toxicity range of casticin in RAW264.7 cells. TRAP staining and F-actin staining determined that casticin can significantly inhibit the differentiation and function of osteoclasts. Then, quantitative polymerase chain reaction (qPCR) and Western blotting further clarified the expression of specific genes and proteins that can inhibit osteoclastogenesis (differentiation and function) through the NF-κB/BCL-2 signaling pathway. These findings aim to enhance the understanding of casticin's molecular mechanism in inhibiting osteoclastogenesis (differentiation and function), potentially paving the way for the development of novel drugs for osteoporosis.

## Methods

### Cell Culture

**Osteoclasts**

Utilizing the RAW 264.7 cells (sources from Shanghai Chuanqiu Biological Company, from ATCC, China), which come from a murine monocyte-macrophage cell line, we were able to study the sequential events leading to OC differentiation induced only by RANKL. The RAW 264.7 cells were cultured in a high-glucose DMEM medium supplemented with 10% fetal bovine serum (FBS, Gibco, USA), 100 U/ml penicillin, and 100 μg/ml streptomycin. The cell cultures were maintained in a cell culture incubator at a constant temperature of 37°C, under 5% CO_2_ and saturated humidity conditions. RAW 264.7 cells in logarithmic growth phase were utilized for experimentation. Specifically, RAW 264.7 cells were seeded into 96- and 24-well plates at a density of 1500 cells per well, and 6-well plates were used for seeding at a density of 3 × 10^4^ cells per well.

### Cytotoxicity Test

RAW 264.7 cells were cultured in 96-well plates with DMEM medium containing 75 ng/ml RANKL (R&D Systems, USA) for 24, 48 and 72 h, respectively. To evaluate the cytotoxicity and impact of casticin on RAW 264.7 cells, different concentrations of casticin (0, 0.125, 0.25, 0.50, 1.00, 2.00, and 4.00 μM) were added to the experimental groups. Subsequently, 96-well plates labeled for 24, 48, and 72 h were subjected to the addition of CCK8 reagent (US EVERBRIGHT, USA) at 10% of the well volume at the designated time points. The plates were then incubated in a CO_2_ cell incubator (Thermo Fisher Scientific, USA) for 2 h. Finally, the absorbance (OD) value of each well was measured using an enzyme-linked immunosorbent assay (Thermo Fisher Scientific), with the wavelength set at 450nm. This assay allowed us to assess the cytotoxicity and viability of RAW 264.7 cells under different casticin concentrations and exposure durations.

### Tartrate-Resistant Acid Phosphatase (TRAP) Staining

In the TRAP staining conducted in 96-well plates, the experimental groups consisted of cells treated with RANKL (75 ng/ml) and casticin at concentrations of 0.125, 0.25, and 0.50 μM, while the control groups were treated solely with RANKL (75 ng/ml). Each group comprised three wells for the analysis. After approximately one week of cell cultivation, during which osteoclasts reached the mature stage, the cells were fixed using 4%paraformaldehyde at room temperature. Following fixation, the cells were first washed three times with a PBS solution. Subsequently, TRAP staining solution (Sigma, USA) was added to the wells. The cells were then stained in a 37°C incubator, shielded from light, for a duration of 30 minutes. Under a light microscope, osteoclasts were identified as TRAP-positive cells with three or more nuclei. This staining process allowed for the visualization and quantification of osteoclasts in the different experimental and control groups.

### Phalloidin Staining

Following treatment with 0.1% (v/v) Triton X-100 for a duration of 5 min, the osteoclasts on glass slides were subjected to thorough washing with PBS solution to minimize residual Triton X-100. Subsequently, the cells on the chamber slides within each well were initially stained with a diluted phalloidin solution (5 μg/ml, Solebao Co., USA) for a period of 60 min under room temperature and in a dark environment. Following the phalloidin staining, the cell slides underwent two washes with PBS. Osteoclasts were then re-stained with a DAPI solution, and this re-staining step lasted for 5 minutes. Subsequently, the DAPI solution was discarded, and the cell slides were washed three times with PBS. The slides were then carefully removed and allowed to dry. To seal the stained samples, a sealing solution containing an anti-fluorescence quenching agent was applied. Finally, the stained osteoclasts were observed and captured using a fluorescence confocal microscope. This staining procedure allowed for the visualization and examination of the actin filaments within the osteoclasts and their nuclei using fluorescent microscopy techniques.

### qPCR

To investigate the impact of different casticin concentrations on the expression of specific genes in osteoclasts, cell cultures were conducted in 6-well plates. The experimental groups consisted of cells treated with RANKL (75 ng/ml) and various concentrations of casticin (0.125, 0.25, 0.50 μM). The control groups were treated with RANKL (75 ng/ml) and cultured for approximately one week. At each stimulation time, cells (treated and untreated) were detached from the wells and washed once with PBS. Total RNA was isolated using the “GenElute Mammalian Total RNA Miniprep Kit” (Sigma-Aldrich, USA) and quantified by using the bio photometer (Eppendorf, Germany). Total RNA (3 μg) was converted to cDNA using the SuperScript Vilo (Quan Shi Jin Biotech Co., Ltd., China), according to the protocol. For the qPCR analysis, 9.2 μl of cDNA containing RNase-Free dH_2_O, 0.4 μl of Forward Primer, 0.4 μl of Reverse Primer, and 10 μl of TOP/TIP Green qPCR SuperMix were combined to form a 20 μl reaction system. The threshold cycle (CT) values were calculated against the housekeeping gene GAPDH. To assess the impact of casticin on the expression of CtsK, c-FOS, MMP-9, and NFATc1 in osteoclast differentiation at various concentrations and time points, the expression levels of the analyzed genes are presented as relative values of the cells treated with casticin compared to the cells not treated with casticin. The qPCR primers were provided by China Huada Gene Co., Ltd., and their sequences were detailed in Table 1.

### Western Blotting

To assess the dose-dependent effects of casticin on the expression of osteoclast-specific proteins, cells were cultured in 6-well plates. Experimental groups were treated with RANKL (75 ng/ml) in combination with casticin at concentrations of 0.125, 0.25, and 0.50 μM, while control groups received RANKL (75 ng/ml) alone. Cells were incubated with the respective treatments for approximately 1 week before protein extraction. Briefly, cells were washed with pre-cooled PBS, followed by the addition of a lysis buffer containing a 2:1:97 volume ratio of protease inhibitors, phosphatase inhibitors, and RIPA lysate. The cells were then lysed on ice for 30 min. Protein concentrations in the lysates were determined using the BCA method. Subsequently, an appropriate amount of protein was subjected to electrophoresis and transferred onto a PVDF membrane. The membrane was blocked with 5% skimmed milk diluted in TBST at room temperature for 1 h with agitation. After blocking, the membrane was incubated overnight at 4°C with specific monoclonal antibodies against c-FOS, NFATc-1, CtsK, MMP-9, and GADPH (rabbit anti-mouse antibodies from CST company in the United States). On the following day, the membrane was washed three times with TBST and then incubated with secondary antibodies at room temperature for 2 h. After three additional washes with TBST, the membrane was exposed and imaged using a gel imager with a highly sensitive chemiluminescence solution.

To further elucidate the molecular mechanisms underlying the impact of casticin on osteoclast differentiation, cells were cultured in 6-well plates. Simultaneously, RAW 264.7 cells were subjected to a 2-h starvation treatment by removing the FBS supplement. Control groups were treated with RANKL (75 ng/ml) for 15, 30, 45, and 60 min, while experimental groups were treated with casticin (0.50 μM) and RANKL (75 ng/ml) for the same durations (15, 30, 45, and 60 min). After 1 h, protein was extracted using the same method as described earlier. Western blotting was employed to assess the levels of p65/p-p65, IκBα/P-IκBα, BCL-2, and GADPH (with no absolute values). Image J software was utilized for the relative quantification of protein bands from Western blot films.

### Statistical Analysis

All experimental data were quantitative data, represented by X ± S, and t-tests were conducted to compare the differences between the two groups. The statistical analysis were performed by SPSS 22.0, and GraphPad Prism 9.0. *P*-value < 0.05 indicated a statistically significant difference, otherwise speified.

## Results

### Determination of the Cytotoxic Range of Casticin on RAW 264.7 Cell

**Fig. 1:**
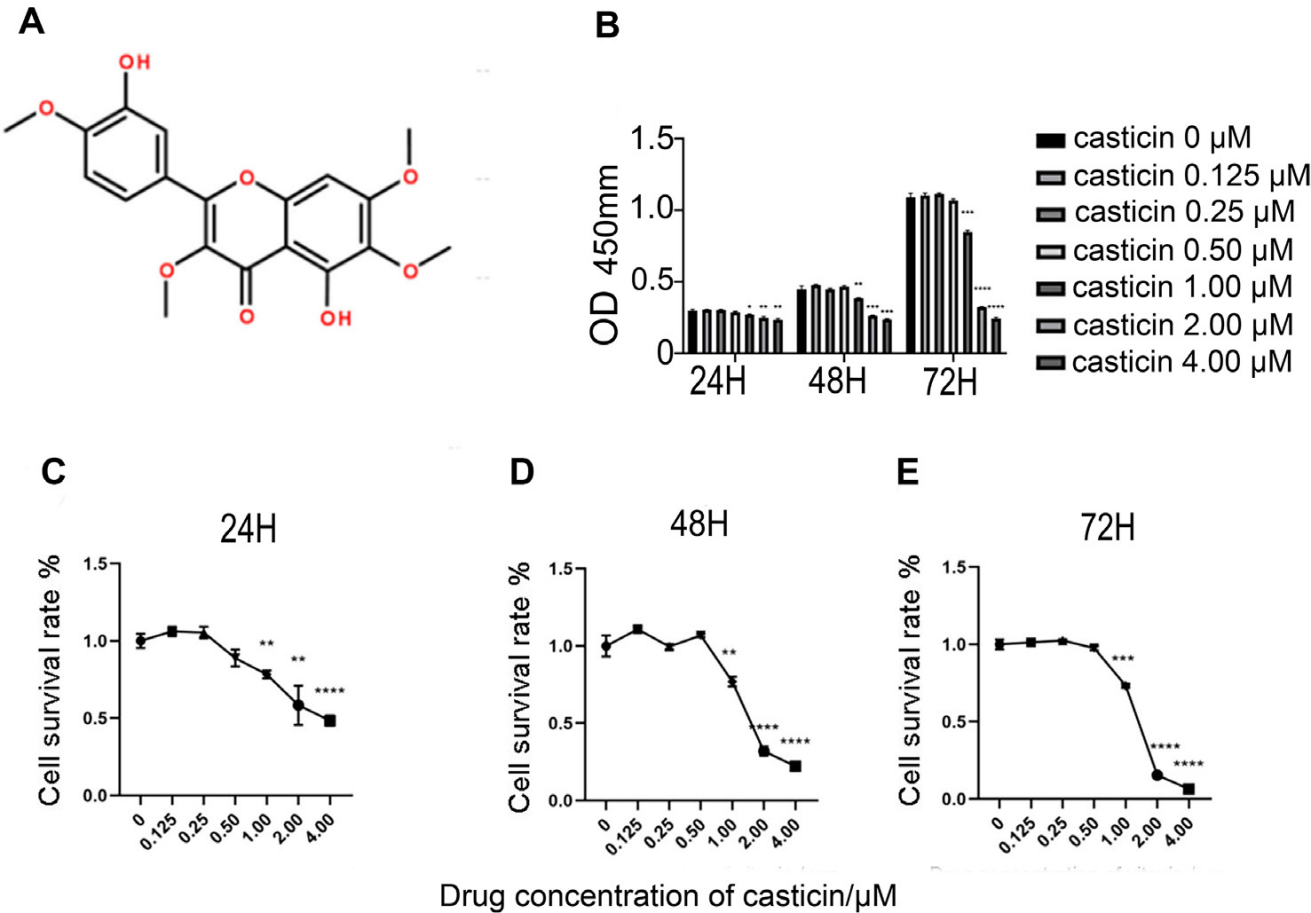
Casticin inhibited the proliferation of osteoclasts in a time and dose dependent manner. (**A**) Chemical molecular structural formula of casticin. (**B**) There was a significant statistical difference in the absorbance values of casticin in each group at 24, 48, and 72 h when the concentration of casticin was ≥ 1 μM. (**C-E**) Represent the cell survival rates at 24, 48, and 72 h, respectively. When the concentration of casticin ≤ 0.50 μM, there was no significant effect on the cell activity of RAW 264.7 cell. The results were consistent with b. (Note: “*” represents a significant statistical difference compared to 0 μM casticin. The more “*”, the greater the statistical difference. *P-value < 0.05, **P-value < 0.01, ***P-value < 0.001, ***P-value < 0.0001.)

The safe medication range for subsequent experiments was determined through exploring the drug toxicity range of casticin on RAW 264.7 cell by CCK8 cytotoxicity experiments. The results showed that the casticin of concentration ≥ 1.00 μM significantly inhibited the activity of RAW 264.7 cell (*p*-value < 0.05, Fig. 1). Therefore, the 0-0.50 μM concentration of casticin was be set at subsequent experiments.

### High Concentration of Casticin Can Inhibit the Differentiation and Function of Osteoclasts

**Fig. 2:**
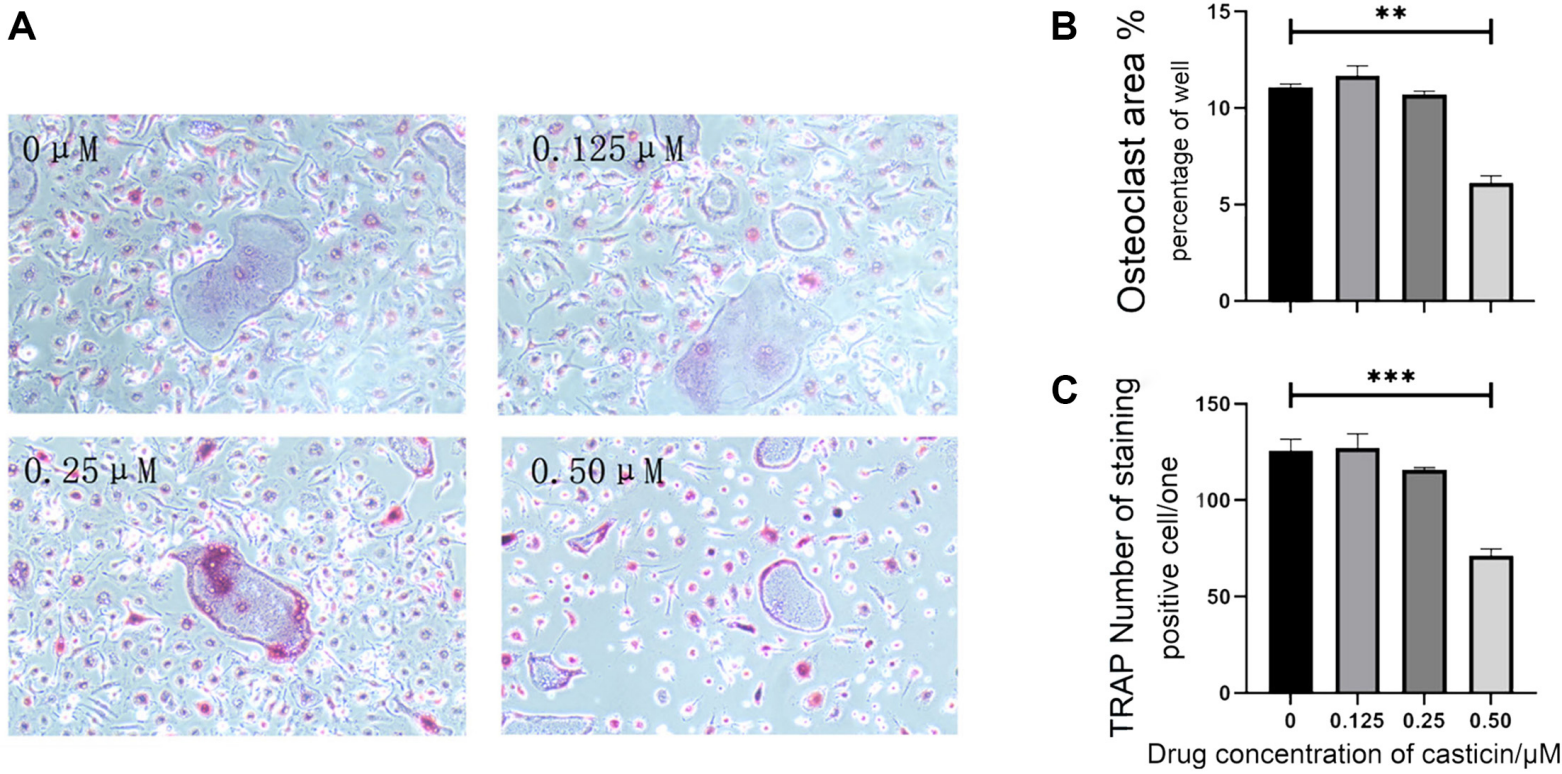
Casticin inhibited the expression TRAP. (**A**) TRAP staining results at different concentrations. (**B**) The percentage of pore area occupied by osteoclasts. (**C**) The number of TRAP staining positive cells. 0.50 μM casticin can significantly inhibit the differentiation of osteoclasts. (Note: “*” represents a significant statistical difference compared to 0 μM casticin. The more “*”, the greater the statistical difference. *P-value < 0.05, **P-value < 0.01, ***P-value < 0.001, ***P-value < 0.0001).

**Fig. 3:**
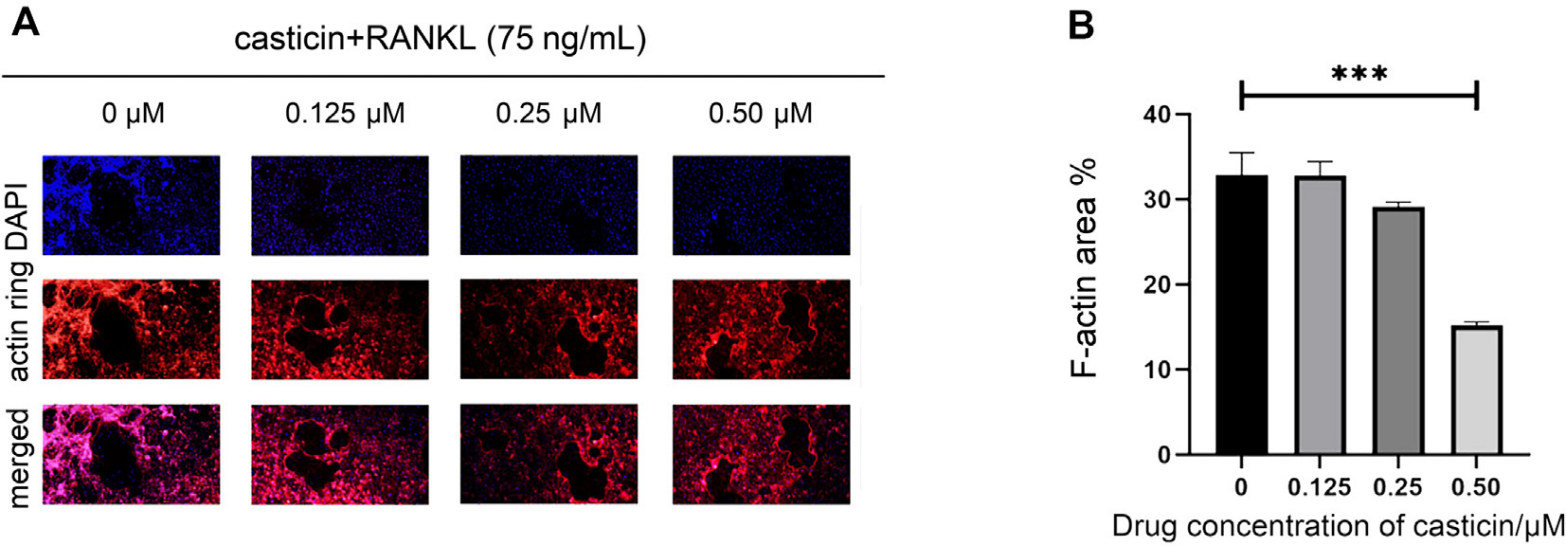
Casticin inhibited the actin filament growth. (**A**) F-actin immune-staining results. (**B**) The ratio of F-actin to the area of each pore showed that casticin could significantly inhibit the area of the actin ring at 0.50 μM. (Note: “*” represents a significant statistical difference compared to 0 μM casticin. The more “*”, the greater the statistical difference. *P-value < 0.05, **P-value < 0.01, ***P-value < 0.001, ***P-value < 0.0001).

TRAP staining results showed that casticin had a mild promoting effect on osteoclast differentiation at 0.125 μM, however, there was no statistically significant difference. At 0.25 μM, mild inhibition of osteoclast differentiation was observed. At 0.50 μM, there was a significant inhibitory effect on the differentiation of osteoclasts (*p*-value < 0.05, Fig. 2). F-actin staining results showed that casticin had no significant effect on the area of the actin ring at 0.125 μM but could slightly reduce the area of the actin ring at 0.25 μM, and significantly reduce the area of the actin ring at 0.50 μM (*p*-value < 0.05, Fig. 3).

### Casticin Decreased the Expression of Osteoclast Marker Genes

**Fig. 4:**
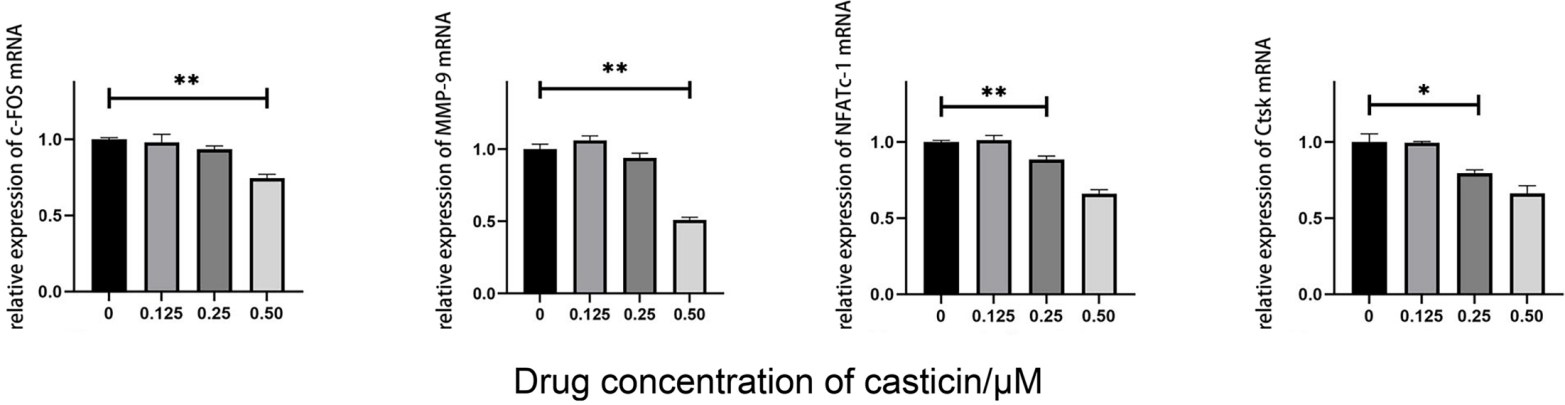
mRNA expression analysis of osteoclast marker genes by q-PCR. 0.50 μM casticin reduces the expression of NFATc-1, c-FOS, CtsK and MMP-9 genes in osteoclasts, with significantly statistical differences. (Note: “*” represents a significant statistical difference compared to 0 μM casticin. The more “*”, the greater the statistical difference. *P < 0.05, **Pvalue < 0.01, ***P-value < 0.001, ***P-value < 0.0001).

**Fig. 5:**
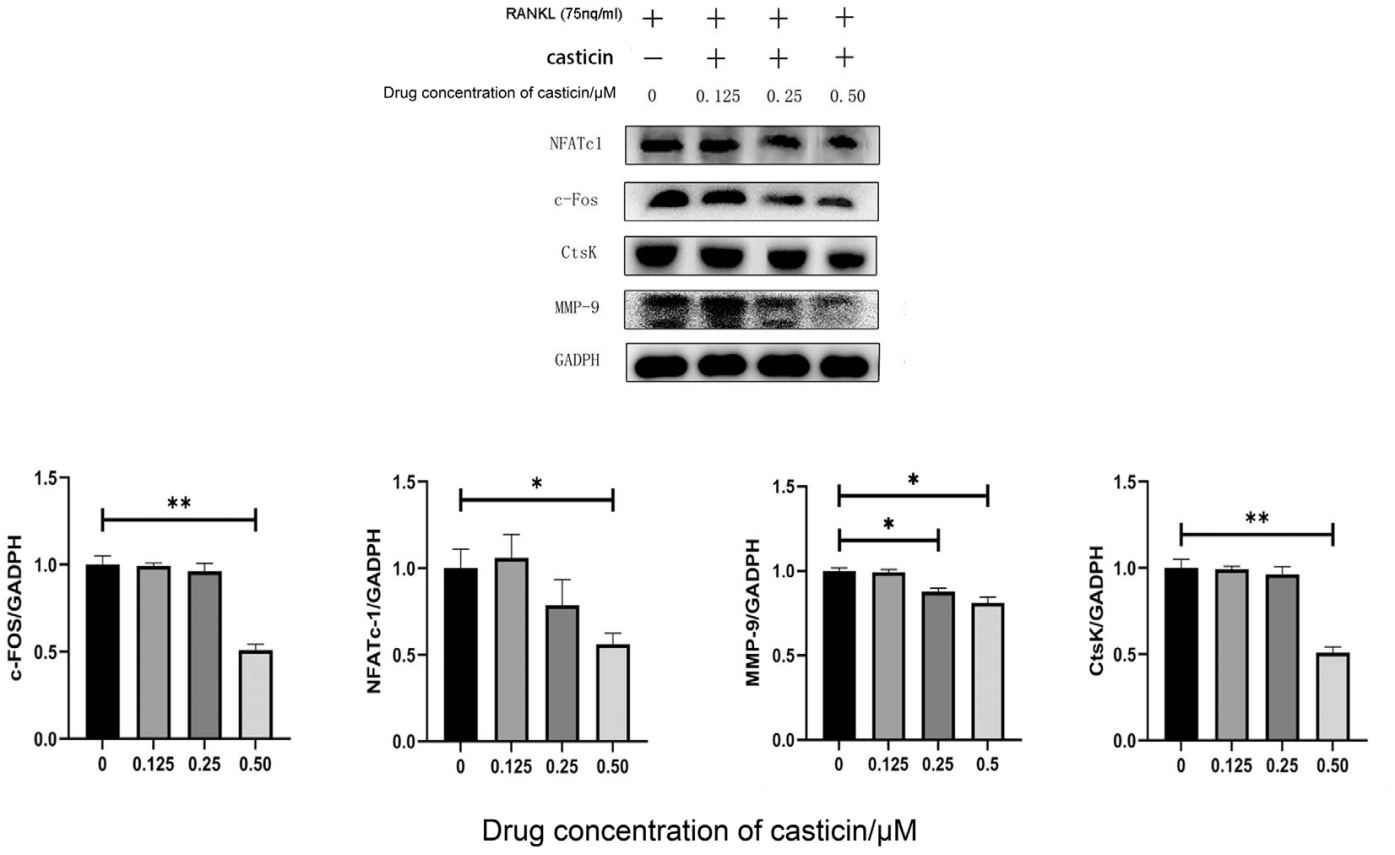
Protein expression analysis of osteoclast marker genes by Western blotting. 0.50 μM casticin reduces the expression of NFATc-1, c-FOS, CtsK and MMP-9 genes in osteoclasts, with significantly statistical differences. (Note: “*” represents a significant statistical difference compared to 0 μM casticin. The more “*”, the greater the statistical difference. *P-value < 0.05, **P-value < 0.01, ***P-value < 0.001, ***P-value < 0.0001).

qPCR and Western blot were used to detect the expression of specific osteoclast markers such as c-FOS, NFATc1, MMP-9 and CtsK proteins. The qPCR results showed that the mRNA levels of c-FOS and NFATc-1 were significantly reduced under the action of 0.50 μM casticin (*p*-value < 0.05), while the mRNA levels of CtsK and MMP-9 were significantly reduced under the action of 0.25 and 0.50 μM casticin (*p*-value < 0.05, Fig. 4). In addition, Western blot results showed that the expression of c-FOS, NFATc-1 and CtsK proteins was significantly reduced under the action of 0.50 μM casticin (*p*-value < 0.05), while the expression of MMP-9 protein was significantly reduced under the action of 0.25 and 0.50 μM casticin (*p*-value < 0.05, Fig. 5). These findings suggested that Casticin inhibited the differentiation of osteoclasts.

### Casticin Inhibited Osteoclasts through the NF-κB/BCL-2 Signaling Pathway

**Fig. 6:**
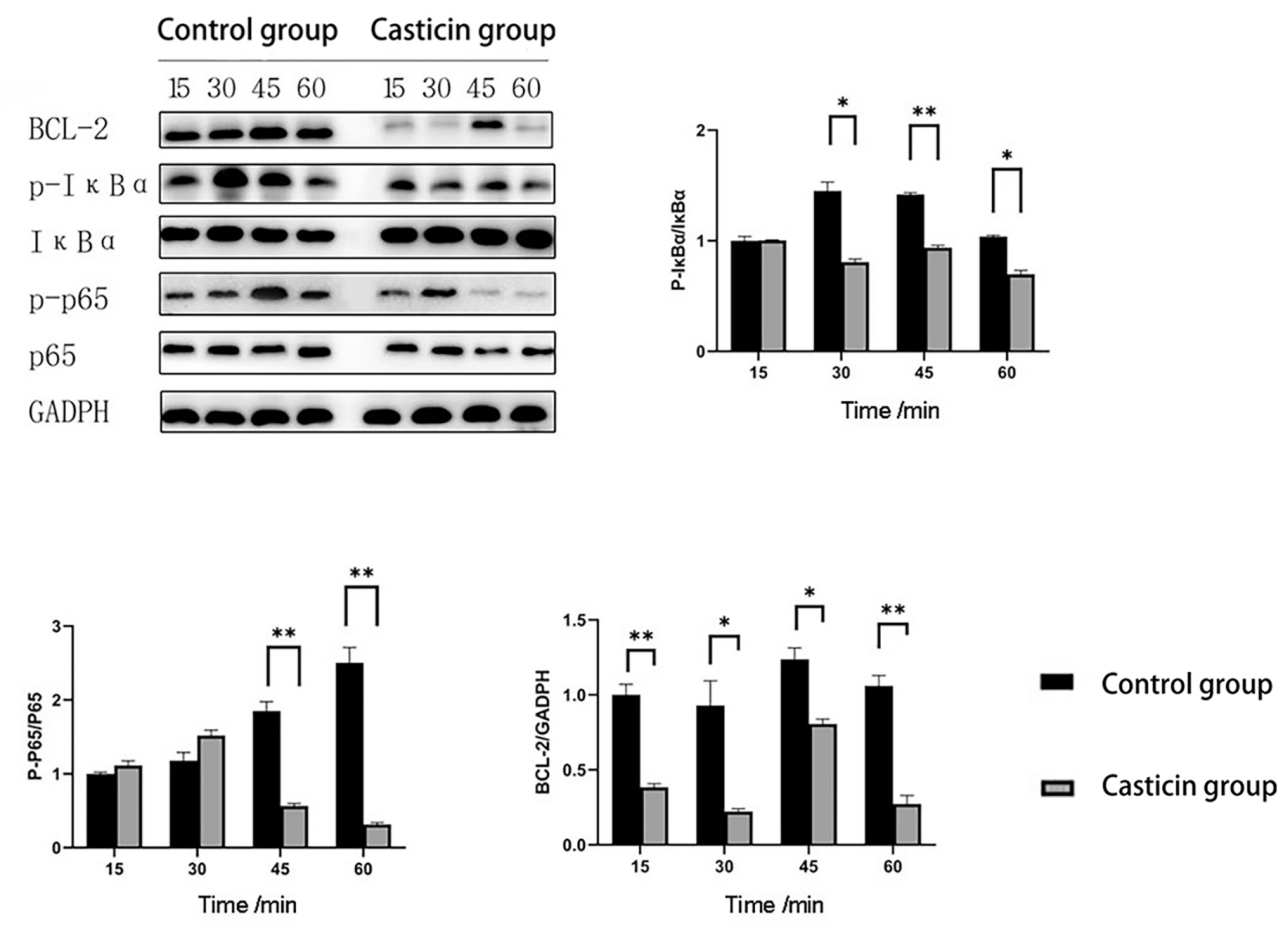
Protein expression analysis of NF-κB/BCL-2 signaling pathway under 0.50 μM casticin treatment. 0.50 μM casticin treatment significantly reduced the expression of proteins such as p65/p-p65, IκBα/p-IκBα and BCL-2 in osteoclasts. (Note: “*” represents a significant statistical difference compared to 0 μM casticin. The more “*”, the greater the statistical difference. *P-value < 0.05, **P-value < 0.01, ***P-value < 0.001, ***P-value < 0.0001).

Western blot was used to detect NF-κB/BCL-2 signaling pathway proteins, including p65/p-p65, IκBα/p-IκBα and BCL-2. The results showed that 0.50μM of casticin had no significant effect on non-phosphorylated p65 and IκBα. In order to further investigate the mechanism of the inhibitory effect of casticin on osteoclasts, the results showed that the ratio of p-p65 to p65 was significantly reduced in the experimental groups compared to the control groups at 45 and 60 min (*p*-value < 0.05), and the ratio of p-IκBα to IκBα was significantly reduced in the experimental groups compared to the control groups at 30, 45 and 60 min (*p*-value < 0.05). Moreover, to determine the expression of the downstream molecule BCL-2 of NF-κB protein, we found that the expression of BCL-2 in the experimental groups was significantly lower than that in the control group at 15, 30, 45 and 60 min (*p*-value < 0.05), (Fig. 6).

## Discussion

Osteoporosis is a systemic metabolic disease partly caused by the dysregulated osteoclastogenesis (differentiation and function) with limited treatment options. This study established the safe range of casticin for RAW 264.7 cells as a preliminary step for subsequent experiments. Casticin exhibited significant inhibition of osteoclastogenesis (differentiation and function) and function at a concentration of 0.50 μM in RAW 264.7 cells. Furthermore, it was observed that casticin (0.50 μM) reduced the expression of osteoclast marker genes such as c-FOS, NFATc1, MMP-9, and CtsK during osteoclastogenesis (differentiation and function). Further exploration into the molecular mechanisms underlying casticin inhibition of osteoclasts revealed that casticin significantly reduced the expression of phosphorylated IκBα and p65, which were consistent with the observed reduced expression of BCL-2 molecules. These findings suggest that casticin may inhibit osteoclastogenesis (differentiation and function) through the NF-κB signaling pathway. This potential mechanism could hold significance of casticin for drug development in osteoporosis.

Our study demonstrated that casticin significantly inhibited the differentiation and function of osteoclasts. Osteoclasts originate from the monocyte-macrophage system and are the sole cells with bone resorption capabilities. Overproduction of osteoclasts can contribute to the development of osteoporosis [13]. Normal bone metabolism relies on a delicate balance involving osteoclasts, responsible for bone resorption. Imbalances in this equilibrium can lead to pathological or inflammatory bone diseases [15]. Notably, casticin exhibits anti-inflammatory properties [16]. These findings suggest that casticin may inhibit osteoclasts through its anti-inflammatory effects.

We also found that casticin downregulated the expression of specific genes and proteins including c-FOS, NFATc1, MMP-9, and CtsK during osteoclastogenesis (differentiation and function). The expression of c-FOS activates NFATc1, a pivotal transcription factor in osteoclastogenesis (differentiation and function), regulated by phosphorylation via distinct NFAT kinases like casein kinase 1 (CK1) [17]. Clinical observations revealed elevated circulating MMP-9 levels in patients, particularly those with osteoporosis, suggesting MMP-9 as a potential biomarker for increased bone resorption [18]. Cathepsin K, a cysteine protease highly expressed by activated osteoclasts, plays a crucial role in degrading type I collagen, the primary component of the bone matrix. Given its pivotal role in the initial stages of bone resorption, cathepsin K has emerged as a therapeutic target in osteoporosis [19]. Mature osteoclasts secrete osteoclast-specific proteins like MMP-9 and CtsK, contributing to bone resorption [20, 21]. Collectively, inhibiting their function by casticin holds promise for preventing and treating osteoporosis.

Further investigation into the molecular mechanism of casticin's inhibitory effect on osteoclasts revealed its significant suppression of phosphorylated IκBα and NF-κB. This phenomenon may be attributed to the stable binding of non-phosphorylated i-κB α to NF-κB, preventing phosphorylation and translocation of p65, which is consistent with our experimental results that cricin inhibits the expression of phosphorylated p65 and IκBα, thereby reducing the ratio of phosphorylation to non-phosphorylation. The observed reduction in NF-κB activation correlated with decreased downstream expression of BCL-2, consistent with the significantly reduced and statistically different BCL-2 expression in this study. In the process of osteoclast formation, RANKL and M-CSF initially activate the NF-κB and mitogen-activated protein kinase (MAPK) signaling pathways, subsequently triggering c-FOS and NFATc-1 activation. NF-κB is ubiquitous in animal cell nuclei, remaining inactive in an unstimulated state [22, 23]. Upon phosphorylation of IκBα, NF-κB becomes active, translocating into the nucleus to bind to NF-κB responsive elements, thereby promoting target gene transcription [24, 25]. Furthermore, studies have indicated that upon cellular stimulation, nuclear NF-κB further regulates the transcription of apoptosis-related gene BCL-2, influencing cell differentiation, proliferation, and apoptosis [26]. This highlights BCL-2 as one of the downstream molecules of NF-κB., and according to our signaling pathway experiments indicated that casticin can significantly inhibited the expression of phosphorylated p65 and IκBα, thereby reducing the ratio of phosphorylation to non-phosphorylation. Additionally, NF-κB inhibition was associated with a significant reduction in BCL-2 expression, Therefore, it is further demonstrated that BCL-2 is a downstream molecule of NF-κB.Investigating how casticin regulates the NF-κB/BCL-2 signaling pathway in osteoclasts represents a promising direction for future studies and potential clinical applications.

There are limitations to this study. The NF-κB/BCL-2 signaling pathway plays a crucial role in osteoclastogenesis (differentiation and function), as demonstrated in numerous studies. Later, we conducted *in vitro* cell experiments based on the mouse model of postmenopausal osteoporosis to further improve the effect of casticin on osteoclast generation in animals through NF-κB/BCL-2 signaling pathway. In summary, our study suggests that casticin effectively inhibits osteoclastogenesis (differentiation and function) and maturation by regulating the NF-κB/BCL-2 signaling pathway. These findings highlight the great potential of cricin as a Traditional Chinese Medicine-based treatment for postmenopausal osteoporosis.

## Figures and Tables

**Table 1:** Primer sequences of osteoclast marker genes.

Genes	Primer sequence (Forward 5’-3’)	Primer sequence (Reverse 5’-3’)
Fos	AGGCAGAACCCTTTGA	GGTGACCACGGGAGTA
NFATc-1	TCTTCCGAGTTCACATCCC	GACAGCACCATCTTCTTCC
CtsK	GAAGAAGACTCACCAGAAGCAG	TCCAGGTTATGGGCAGAGATT
MMP-9	CTGGACAGCCAGACACTAAAG	CTCGCGGCAAGTCTTCAGAG
GADPH	AACGACCCCTTCATTGACCTC	CCTTGACTGCCGTTGAACT
